# Brain correlates of cognitive reserve in offspring of patients with schizophrenia or bipolar disorder

**DOI:** 10.1007/s00787-026-03071-x

**Published:** 2026-06-27

**Authors:** Patricia Camprodon-Boadas, Elena De la Serna, María Ortuño, Daniel Ilzarbe, Adriana Fortea, Inmaculada Baeza, Roger Borras, Carla Torrent, Irene Martinez-Serrano, Clemente García-Rizo, Dolores Moreno, Josefina Castro-Fornieles, Gisela Sugranyes

**Affiliations:** 1 Department of Child and Adolescent Psychiatry and Psychology, 2021SGR01319, Institut Clinic de Neurociències, Hospital Clínic Barcelona, Barcelona, Spain; 2 Fundació de Recerca Clínic Barcelona-Institut d’Investigacions Biomèdiques August Pi i Sunyer (IDIBAPS), Barcelona, Spain; 3 Centro de Investigación Biomédica en Red de Salud Mental (CIBERSAM), Instituto de Salud Carlos III, Madrid, Spain; 4 Department of Medicine, Institute of Neurosciences, University of Barcelona, Barcelona, Spain; 5 Barcelona Bipolar and Depressive Unit, Institute of Neurosciences, Barcelona, Spain; 6 Barcelona Clínic Schizophrenia Unit, Neuroscience Institute, Hospital Clínic, Barcelona, Spain; 7 Department of Child and Adolescent Psychiatry, Hospital Universitario Sureste, Madrid, Spain; 8 Department of Child and Adolescent Psychiatry, Institute of Psychiatry and Mental Health, Marañón School of Medicine, Hospital General Universitario Gregorio, Universidad Complutense, Madrid, Spain

**Keywords:** Cognitive reserve, Schizophrenia, Bipolar disorder, Magnetic Resonance Imaging

## Abstract

Cognitive reserve (CR) modulates the impact of neural alterations on clinical outcomes, yet its association with brain structure during youth remains unexplored. We aimed to investigate the relationship between CR and brain structure in young offspring of patients with schizophrenia or bipolar disorder, according to development of psychotic spectrum symptoms over time. 404 data acquisitions were analyzed from offspring of patients with schizophrenia or bipolar disorder, 43 offspring who developed psychotic spectrum symptoms over time (SzBpO-P), 94 offspring who did not (SzBpO-NP), and 80 community controls (CcO), re-assessed over a 12-year period (101 SzBpO-P, 181 SzBpO-NP, 122 CcO). Global and lobar volume, cortical surface area and thickness metrics were obtained from T1-weighted sequences acquired on a 3 Tesla MRI scanner and computed using FreeSurfer version 7.1. CR was calculated using a composite score including an estimated intelligence quotient, sociability, parental socioeconomic status, school performance, and developmental milestones. Multilevel mixed-effect logistic regression (categorical variables) or linear regression (continuous variables) models were conducted. A significant group-by-CR interaction was found for total intracranial volume (F = 7.770; *p* < 0.001), whereby the CcO and SzBpO-NP groups showed a positive association between CR and total intracranial volume, while this association was negative for SzBpO-P. No significant effects were observed in other brain structural metrics when adjusting for total intracranial volume. CR was differentially associated with total intracranial volume according to development of psychotic spectrum symptoms. These results may help identify youth at highest risk for severe mental health outcomes.

**Supplementary Information:**

The online version contains supplementary material available at https://doi.org/10.1007/s00787-026-03071-x.

## Introduction

Accumulating evidence has demonstrated that schizophrenia and bipolar disorder exhibit shared genetic [1], clinical [2], and brain structural features [3]. Both disorders are characterized not only by high heritability [1], but also by cross-heritability, whereby relatives of individuals diagnosed with either schizophrenia or bipolar disorder are at increased risk of both disorders [1, 4]. Offspring of patients with schizophrenia (SzO) and bipolar disorder (BpO) are considered to be at heightened risk of both disorders due to a combination of genetic and environmental factors. Compared to offspring of individuals without these diagnoses, SzO and BpO show higher lifetime rates of any axis I disorder [5–7], poorer psychosocial functioning, and smaller brain volumes, especially in those who develop psychotic spectrum symptoms at follow-up [8]. Notably, these effects tend to be more pronounced in SzO than in BpO [6, 9].

There is evidence of disease specificity in the brain structural correlates of familial risk for schizophrenia and bipolar disorder during childhood and adolescence [10], whereby SzO are characterized by smaller global and regional cortical grey matter volumes, while young BpO as a group show no differences in these measures relative to controls. However, our group found that BpO and SzO who develop psychotic spectrum symptoms over time (SzBpO-P) exhibit overlapping changes in brain structure relative to those who do not display symptoms (SzBpO-NP), consisting of greater cortical thinning over time and smaller cortical surface area [8]. These findings mirror evidence of brain structural changes in individuals with psychosis and at clinical high risk for the disease [11, 12]. Childhood and adolescence is an especially valuable period for assessing changes in brain structure associated with risk for disease, since it coincides with a key period in brain structural maturation, for instance, global brain volumes reach their peak between ages 10 and 15 [10, 13, 14].

Research priorities in psychiatry have begun to shift from the field of risk to the field of protective factors. Cognitive reserve (CR) was coined in the field of neurology and defined as the inter-individual capacity to cope with age- or pathology-related brain alterations [15, 16], which allows individuals with higher CR to endure more advanced stages of disease before exhibiting symptoms [15]. The most commonly used variables for measuring CR are intelligence, educational and occupational level, and engagement in leisure and social activities [17, 18]. In psychiatry, most studies assessing CR so far have focused on adult patients experiencing first-episode psychosis or bipolar disorder [17–19]. Only one study to date has examined CR in young SzO and BpO, and found lower CR levels in SzO than controls, with BpO at an intermediate level [20]. Higher CR was associated with fewer attenuated psychotic symptoms and better psychosocial functioning, in all groups, but strongest in SzO [20]. However, this previous work was limited by a relatively small sample size and did not include neuroimaging measures.

To the best of our knowledge, no studies so far have investigated the relationship between CR and brain structure in children and adolescents. In healthy adults, CR has been found to be positively correlated with global and regional gray matter volumes [21–23], while in individuals in the early stages of Alzheimer’s disease or mild cognitive impairment, CR has been found to correlate negatively with brain volumes [24]. This has been interpreted as a compensatory phenomenon [25, 26], in which higher CR could reflect the brain’s ability to use pre-existing or new strategies to maintain functioning, despite structural decline [27, 28]. We aimed to investigate the association between CR and brain structure in SzO and BpO, grouped based on the development of psychotic spectrum symptoms over time, compared to community control offspring (CcO). In the absence of prior evidence in youth and given that in adults with Alzheimer’s disease the relationship between CR and brain structure follows a stage-based pattern, we aimed to extend this framework to the early stages of psychosis. First, we expected to find lower CR in SzBpO, especially in those who developed psychotic spectrum symptoms over time, and a positive relationship between CR and psychosocial functioning for the overall sample. We then hypothesized that higher CR would be associated with larger brain volume in CcO and in SzBpO-NP, but that this association would be inversed in SzBpO-P. As secondary analyses we tested whether our findings differed according to parental diagnosis (schizophrenia versus bipolar disorder) and structural brain metrics (global versus regional brain volumes, cortical thickness and surface area).

## Methods

### Participants

The BASYS cohort is located at the Child and Adolescent Psychiatry and Psychology Departments of the Hospital Clinic Barcelona and Hospital Gregorio Marañón Madrid, Spain, with local ethics review board approval. The present study used data from the Barcelona site only, in order to minimize MRI scanner related confounds. All participants provided written informed consent. For participants under age 18, written informed consent was obtained from participants and their parents or legal guardians.

Patients diagnosed with schizophrenia or bipolar disorder at the Department of Psychiatry and Psychology of the Hospital Clinic Barcelona, with offspring aged 6–17 years were invited to participate. Control parents were recruited from the same geographical area as the patients. CcO were aged 6 to 17 years and did not have a first or second-degree family history of schizophrenia or bipolar disorder. For further details regarding the recruitment and evaluation of BASYS see [7].

Exclusion criteria for all groups: intellectual disability, significant head injury and a history of severe medical or neurological conditions. Offspring scoring 3 or more in any item of the positive and negative subscales [29] of the Scale Of Prodromal Symptoms [30] at any timepoint were classified as presenting psychotic spectrum symptoms. In these cases, all available assessments from that participant were assigned to the SzBpO-P group. This classification included both individuals with attenuated (scores between 3 and 5) and clinical (score of 6) psychotic symptoms (*n* = 5 met criteria for psychosis not otherwise specified and *n* = 2 for bipolar disorder). Fourty-three SzBpO-P, 94 SzBpO-NP, and 80 CcO were included in the study. Participants were matched for age and sex. Families were contacted for follow-up visits at up to two, four, eight, and twelve years, in which all assessments were repeated. This yielded a total of 404 datapoints (101 datapoints for SzBpO-P, 181 for SzBpO-NP, 122 for CcO). Given the naturalistic design of the study, cases have different follow-up periods. Details on the number of participants included at each timepoint, those with pending assessments, comparisons between participants who attended only once vs. those who completed follow-up visits, as well as the degree of relatedness between participants, are detailed in the supplementary material (Figure S1, Table S1, Table S2). Sub-analyses of global brain volumes were also conducted within offspring subgroups.

### Clinical assessment

Psychiatrists and psychologists with experience assessing children and adolescents conducted all assessments, using the Kiddie Schedule for Affective Disorders and Schizophrenia, Present and Lifetime version (K-SADS-PL) [31] or the Structured Clinical Interview for DSM-IV Disorders (SCID-I) [32] for lifetime psychopathology, the Global Assessment of Functioning Scale [33] for psychosocial functioning, and the Structured Interview for Prodromal Symptoms [30] for psychotic spectrum symptoms. See Sect. 2 of supplementary material for further details.

### Cognitive reserve assessment

CR was calculated following an age-adapted approach [20, 34], by including: sociability and peer relationships and school performance (both from the Premorbid Adjustment Scale) [35]; achievement of developmental milestones (parental report on language, reading, writing and motor skills, scored on a Likert scale from 0 - abnormal development, to 3 - typical development); parental socio-economic status (Hollingshead-Redlich scale) [36]; academic attainment (whether the participant’s educational level corresponded to their chronological age); and vocabulary sub-test of the Wechsler Intelligence Scale for Children IV (WISC-IV) [37] or Wechsler Adult Intelligence Scale (WAIS-IV) [38]. For further details see supplementary material (Sect. 3, Table S3) and Camprodon-Boadas et al.(2023).

### Image processing

Global measures included total intracranial volume, gray and white matter volume, cortical surface area and thickness, computed using FreeSurfer v.7.1.1 (www.surfer.nmr.mgh.harvard.edu). FreeSurfer automated procedure includes motion correction, non-uniform intensity normalization, registration to Montreal Neurological Institute stereotactic space, skull strip, white matter segmentation, definition of pial surface, and parcellation of the brain into 34 cortical regions per hemisphere using the Desikan-Killiany brain atlas. Regional mean lobar cortical thickness, surface area and grey matter volume per hemisphere were computed and divided into frontal, cingulate, temporal, parietal, occipital, and insular parcellations. Quality assessment was conducted following ENIGMA standard guidelines (https://enigma.ini.usc.edu/protocols/imaging-protocols/) at the level of individual parcellations. If a parcellation failed to comply with quality control criteria, the corresponding lobar measure was not computed for that data point. Additional details about MRI acquisition, sequencing, and processing are provided in Sect. 4 of supplementary material.

### Statistics

To test the normality of the sample distribution, the Kolmogorov-Smirnov test was used, together with Levene’s test to assess the equality of variances.

In order to include a single measure of CR, a factor analysis was conducted with the CcO group at baseline. The analysis extracted three factors and explained 73.2% of the variance. As in previous studies [20, 34, 39], the factorial scores of the three factors were added to obtain a single measure of CR for each participant. For the rest of groups and timepoints, the z-scores of the CR variables were multiplied by the coefficients of the component matrix of the factor analysis conducted with the CcO group at baseline, in order to obtain three factors with the same weight in the same variables. Factorial scores were added to obtain a single measure of CR. A detailed description of the factor analysis can be found in the supplementary material (Sect. 3, Table S3).

To analyze between-group differences in sociodemographic, clinical and psychosocial functioning variables, as well as for CR, multilevel mixed-effect logistic regression models (categorical variables) or multilevel mixed-effect linear regression models (continuous variables) were conducted, with group as fixed factor. To examine the association between CR and psychosocial functioning, a multilevel mixed-effect linear regression model was conducted, with psychosocial functioning as dependent variable, and CR, group and CR-by-group interaction as fixed factors. To test the association between CR and structural brain measures, multilevel mixed-effect linear regression models were conducted with structural brain measures as dependent variable, and CR, group and CR-by-group interaction as fixed factors. All models included age, sex and MRI scanner version (Siemens Trio vs. Prisma) as covariates, and family relatedness (considering subjects who were siblings) and subject ID (accounting for repeated assessments for one individual) as random variables. All analyses were conducted simultaneously for the three groups (SzBpO-P, SzBpO-NP, CcO), except for the analyses for testing the effects of offspring subtypes. All brain volume and surface area measures were performed adjusting for intracranial volume.

The models of global brain measures were repeated with the same covariates and random effects, but with the measure of intelligence quotient (IQ) instead of CR as dependent variable to determine the effect of IQ on brain measures compared to CR. Additional sensitivity analyses were conducted by including lifetime axis I diagnosis, psychotropic medication, and substance use as covariates in order to assess their potential impact on the significant CR, group and CR-by-group interactions. Potential sex effects were examined through a three-way interaction analysis including CR, group and sex on brain structure.

Global and lobar analyses were corrected for multiple comparisons using the Benjamini and Hochberg false discovery rate procedure (FDR, *p* < 0.05) [40], which adjusted for multiple comparisons when testing the effect of CR, the effect of group and the interaction CR-by-group effect separately for global and regional measures independently. All analyses were performed using SPSS version 23 and R version V4.4.0, with bilateral significance set at *p* < 0.05.

## Results

### Demographic and clinical characteristics of the sample

Sociodemographic and clinical characteristics of the sample are detailed in Table 1, Table S6, Table S7. No differences in age, sex, psychotropic medication use, or MRI scanner were observed at any timepoint. The SzBpO-P group exhibited a higher prevalence of lifetime axis I diagnosis compared to the CcO group, and poorer psychosocial functioning and greater severity of attenuated psychotic symptoms compared to the SzBpO-NP and CcO groups. The SzBpO-NP group exhibited lower psychosocial functioning than CcO. See Tables S8 and Table S9 for descriptive characteristics of the sample divided according to offspring subtypes.

**Table 1:** Demographic and clinical information of the sample across all timepoints

	SzBpO-NP	SzBpO-P	CcO	F	*p*	SzBpO-P vs. CcO	SzBpO-NP vs. CcO	SzBpO-P vs. SzBpO-NP
	*N* (%) or Adjusted Mean (SE)					Post-hoc^b^		
Age	14.36 (0.38)	14.36 (0.43)	14.94 (0.44)	1.058	0.348			
Sex: female^a^	88 (48.61)	43 (42.57)	60 (42.48)	0.413	0.662			
Lifetime axis I diagnosis^a^	100 (55.24)	74 (73.26)	52 (42.62)	10.084	**< 0.001**	**0.009**	0.062	0.082
Psychotropic medication ^a^	7 (3.86)	16 (15.84)	1 (0.81)	8.566	**< 0.001**	Ns after Bonferroni correction		
Substance use ^a^	11 (6.07)	11 (10.89)	11 (9.01)	1.278	0.280			
GAF	81.16 (0.84)	72.77 (1.12)	86.87 (1.03)	42.551	**< 0.001**	**< 0.001**	**< 0.001**	**< 0.001**
MRI machine model: Trio^a^	135 (74.58)	80 (79.20)	75 (61.47)	4.848	**0.008**	Ns after Bonferroni correction		
CR	−1.25 (0.25)	−1.81 (0.33)	0.48 (0.30)	14.937	**< 0.001**	**< 0.001**	**< 0.001**	0.542

*CcO* Offspring of community controls, *CR *Cognitive reserve, *GAF *Global Assessment of Functioning, *MRI *magnetic resonance imaging, *SE *standard error, *SzBpO-NP *Schizophrenia and bipolar disorder offspring with psychotic spectrum symptoms, *SzBpO-NP *Schizophrenia and bipolar disorder offspring without psychotic spectrum symptoms, *Ns *non-sigificant. ^a^Multilevel mixed-effect logistic regression models. ^b^Bonferroni multiple comparisons post-hoc

## CR group effects and relationship with psychosocial functioning

There were significant group effects on CR (F = 14.937; *p* < 0.001), whereby SzBpO-P (*p* < 0.001) and SzBpO-NP (*p* < 0.001) had lower CR scores than CcO (Figure S2). No significant differences were found between SzBpO-P and SzBpO-NP (*p* = 0.542). Higher CR was significantly associated with better psychosocial functioning, when assessing the entire sample (F = 77.403; *p* < 0.001) and within each group (all *p* < 0.001, see supplementary material Sect. 7). There was no significant CR-by-group interaction on psychosocial functioning (F = 0.400; *p* = 0.671).

###  Effect of CR-by-group interaction on global MRI measures

**Fig. 1:**
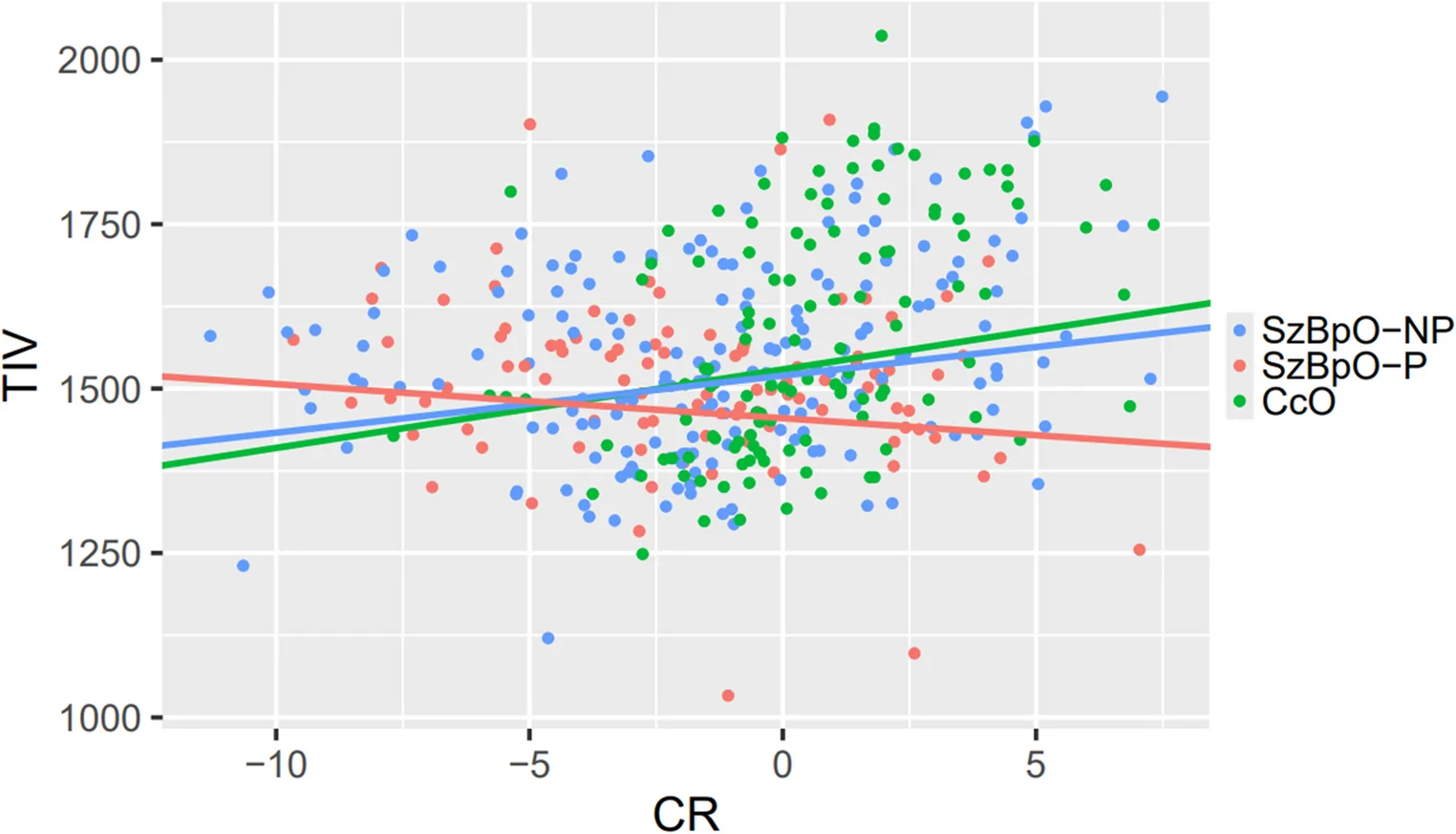
CR-by-group interaction on intracranial volume. CcO = Offspring of community controls; CR = Cognitive reserve; SzBpO-NP = Schizophrenia and bipolar disorder offspring with psychotic spectrum symptoms; SzBpO-NP = Schizophrenia and bipolar disorder offspring without psychotic spectrum symptoms; TIV = Total intracranial volume

There was an effect of group on total intracranial volume (F = 11.870; *p* < 0.001), whereby SzBpO-P showed a significantly smaller total intracranial volume compared to both SzBpO-NP (*p* = 0.001) and CcO (*p* = 0.001). No differences were observed between SzBpO-NP and CcO groups. Group analyses for global MRI measures including total intracranial volume as covariate are provided in Table S10. There was a significant CR-by-group interaction on total intracranial volume (F = 7.770; *p* < 0.001). In SzBpO-NP (β = 13.858; 95% CI = 6.15 to 21.55; *p* < 0.001) and CcO (β = 17.110; 95% CI = 7.02 to 27.19; *p* = 0.001), a positive association was observed, whereby higher levels of CR were associated with larger total intracranial volume compared to the SzBpO-P group. No significant differences were found between the CcO and SzBpO-NP groups. These associations are graphically represented in Fig. 1.

### Effect of CR-by-group interaction on lobar surface area, gray matter volume and cortical thickness measures

No significant CR-by-group interactions were observed on lobar measures of gray matter volume, cortical surface area or thickness.

###  Effect of CR-by-group interaction on global MRI variables on the sample subdivided by offspring subgroups

In SzO, there was a significant CR-by-group interaction on total intracranial volume (F = 7.583; *p* = 0.001). In CcO (β = 17.462; 95% CI = 5.69 to 29.22; *p* = 0.004) and SzO-NP (β = 23.145; 95% CI = 10.43 to 35.85; *p* < 0.001) a positive association was observed, whereby higher levels of CR were associated with larger total intracranial volume compared to the SzBpO-P group. No significant differences were found between the SzO-NP and CcO groups. No other significant CR-by-group interactions were found in global brain structural measures. These results are presented in Table S11 and graphically represented in Figures S3.

In BpO, a significant CR-by-group interaction was observed in total intracranial volume (F = 5.142; *p* = 0.006). In CcO (β = 23.512; 95% CI = 8.75 to 38.27; *p* = 0.002) and BpO-NP (β = 19.922; 95% CI = 6.53 to 33.31; *p* = 0.004) a positive association was observed, whereby higher levels of CR were associated with larger total intracranial volume compared to the SzBpO-P group.No significant differences were found between the BpO-NP and CcO groups. No further significant CR-by-group interactions were found in the other global brain structural measures. These results are presented in Table S12 and graphically represented in Figure S4.

### Sensitivity analyses

Results of sensitivity analyses are presented in supplementary material Sect. 10. The IQ-by-group interaction was significant on intracranial volume (F = 5.767; *p* = 0.003), with the SzBpO-P group exhibiting a negative association between IQ and total intracranial volume, in contrast with the CcO and SzBpO-NP groups. There was no significant effect of lifetime axis I diagnosis, psychotropic medication, and substance use on any of the above findings, both for the entire sample and when analyzed by offspring subgroups. The three-way interaction including CR, group and sex was not significant. The distribution of CR, intracranial volume and white matter volume across groups by sex is presented in Sect. 11 of the supplementary material.

## Discussion

To our knowledge, this is the first study so far to explore the relationship between CR and structural brain measures in children and adolescents at familial high-risk for schizophrenia or bipolar disorder. We found that CR was associated with total intracranial volume across the whole sample, however the direction of this relationship varied in SzO and BpO according to presentation of psychotic spectrum symptoms over time.

In CcO, as well as in SzBpO-NP, higher CR was associated with larger total intracranial volumes. This has also been reported in healthy adults [22, 23, 25], and supports the suggestion that CR may exert a neuroprotective effect on brain structure. Our findings extend this evidence to children and adolescents. Conversely, in SzBpO-P, higher CR was associated with smaller total intracranial volume. A similar effect has been documented in neurodegenerative conditions such as mild cognitive impairment and mild to severe Alzheimer’s disease [24, 26], and has been attributed to a potentially compensatory mechanism of CR in the context of brain damage [28]. This compensatory mechanism is thought to reflect the brain’s resources to maintain function despite structural decline. Higher CR has been associated with better outcomes in SzO and BpO, as well as in individuals with a first-episode psychosis [20, 34], and across all groups in our sample, a greater CR was associated with better psychosocial functioning. Our findings suggest that in SzBpO-P, higher CR, while associated with better psychosocial functioning, was also associated with smaller total intracranial volume, indicating that higher levels of CR could potentially help those with smaller total brain volumes maintain similar levels of functioning [27, 28]. Notably, the significantly smaller total intracranial volume observed in SzBpO-P compared to the other two groups is consistent with studies reporting greater cortical thinning and reduced gray matter volumes in SzBpO-P [8].

Research in animal models of neurodegenerative diseases has shown that CR, conceptualized as an enriched environment, operates through underlying biological mechanisms at both molecular and cellular levels, delaying the onset or progression of disease-related pathology [41]. It has also been suggested that neuroprotective mechanisms could play a role in healthy individuals and in those at the earliest stages of the disease, while compensatory mechanisms become more significant as the disease progresses [25, 28]. The pattern of association between CR and brain structure documented in our sample mirrors this stage-based model described in Alzheimer’s disease [24], which replicates the inverted U theoretical model of CR proposed by Menardi et al. [26]. Recent studies have broadened this perspective by examining the role of CR, not only in individuals diagnosed with Alzheimer’s disease, but also in those at earlier stages of the disease, including pre-clinical stages, high-risk individuals, and those experiencing healthy aging [25, 26, 28]. This conceptual framework emphasizes a stage-based approach to understanding the relationship between CR and brain structure [26], which we have extended to the early stages of psychosis during childhood and adolescence.

The significant association between CR and total intracranial volume, but not with regional brain measures, may reflect the global nature of CR and its potentially broad impact on brain structure. Intracranial volume captures cumulative brain growth, integrating multiple structural components, and is less susceptible to localized variations, making it a broader marker of neurodevelopmental processes [10]. Most components of CR, such as social engagement [42], years of education [43], parental socioeconomic status [44], peer relationships [45], academic achievement [46], and IQ [47] have been independently associated with structural brain metrics. On the other hand, all factors included in our CR proxy are considered to be largely shaped by environmental factors, with the exception of IQ, which represents a highly heritable trait predominantly influenced by genetics [48]. Although both CR and IQ influenced brain measures, the magnitude of the association was stronger for CR, underscoring the contribution of other, potentially modifiable lifestyle factors, in the association between CR and brain volume. This raises the opportunity that by enhancing CR one can impact brain structural development and therefore improve outcomes in vulnerable youth [49].

Our study presents several limitations and strengths that warrant consideration. First, although data were collected at multiple timepoints, the cross-sectional design restricts our ability to establish causality between CR and brain structural measures. In addition, although our CR proxy was comprehensive, it did not include physical exercise or diet, which may also play a role in shaping CR [50]. Moreover, despite our large sample size, the relatively small number of offspring who developed psychotic spectrum symptoms over time may have limited statistical power. Larger studies could allow to stratify more finely according to age, sex, or clinical status. Furthermore, MRI scans were not always acquired during periods of acute symptom expression, which may have reduced sensitivity to detect symptom-related effects on brain metrics. On the other hand, the study’s strengths lie in its considerable sample size given the recruitment method and rich phenotyping of participants, and the innovative application of a hypothesis of CR which was originally coined in a very different field and age range. This adaptation of the CR hypothesis using neuroimaging data in youth at high risk for schizophrenia or bipolar disorder provides insights on potential protective mechanisms explaining differences in clinical outcome and brain structure in this population. Moreover, the inclusion of children and adolescents offers a unique opportunity to study the brain during a critical neurodevelopmental period which is considered key to understanding pathways leading to psychosis. These strengths collectively contribute to advancing our understanding of the role of CR in brain development and its implications for clinical outcomes in psychiatric populations.

In conclusion, we confirm an association between CR and brain structure in offspring of patients with schizophrenia or bipolar disorder according to the presence of psychotic spectrum symptoms. This suggests that the effects of CR on brain structure may follow a stage-based approach depending on the clinical outcome [26]. These findings shed light on potential avenues for intervention aimed at boosting protective mechanisms which may mitigate the impact of familial predisposition to schizophrenia and bipolar disorder on brain development.

## Supplementary Information

Below is the link to the electronic supplementary material.

Supplementary Material 1 (DOCX 2.58 MB)

## Data Availability

Data will be made available upon reasonable request. Only a subset of the data, excluding identifiable individual-level information will be shared.
