# Supplementary material for: Brain correlates of cognitive reserve in offspring of patients with schizophrenia or bipolar disorder
Source: Eur Child Adolesc Psychiatry. 2026 Jun 27;35(11):3487–95. doi: 10.1007/s00787-026-03071-x (PMC13710428; doi:10.1007/s00787-026-03071-x)
Supplement: Supplementary file 1 [file 787_2026_3071_MOESM1_ESM.docx]

**Supplementary material**

**Brain correlates of cognitive reserve in offspring of patients with schizophrenia or bipolar disorder**

European Child & Adolescent Psychiatry

Patricia Camprodon-Boadas, PhD^1,2,3,4*^; Elena De la Serna, PhD^1,3*^; María Ortuño^2^; Daniel Ilzarbe, PhD^1,2,3^; Adriana Fortea, PhD^1,2,3^; Inmaculada Baeza, PhD^1,3,4^; Roger Borras,^2,3^; Carla Torrent, PhD^2,3,4,5^; Irene Martinez-Serrano^2^; Clemente García-Rizo, PhD^2,3,6^; Dolores Moreno, PhD^3,7,8^; Josefina Castro-Fornieles, PhD^1,2,3,4^**; Gisela Sugranyes, PhD^1,2,3^**

^1^ Department of Child and Adolescent Psychiatry and Psychology, 2021SGR01319, Institut Clinic de Neurociències, Hospital Clínic Universitari, Barcelona, Spain.  ^2^ Fundació de Recerca Clínic Barcelona-Institut d´Investigacions Biomèdiques August Pi i Sunyer (IDIBAPS), Barcelona, Spain.^3^ Centro de Investigación Biomédica en Red de Salud Mental (CIBERSAM), Instituto de Salud Carlos III, Madrid, Spain. ^4^ Department of Medicine, Institute of Neurosciences, University of Barcelona, Spain. ^5^ Barcelona Bipolar and Depressive Unit, Institute of Neurosciences, Barcelona, Spain. ^6^ Barcelona Clínic Schizophrenia Unit, Neuroscience Institute, Hospital Clínic, Barcelona, Spain. ^7^Department of Child and Adolescent Psychiatry, Hospital Universitario Sureste, Madrid, Spain. ^8^Department of Child and Adolescent Psychiatry, Institute of Psychiatry and Mental Health, Hospital General Universitario Gregorio Marañón School of Medicine, Universidad Complutense, Madrid, Spain.

*PC and ES contributed equally to this work (co-first authors).

**JC and GS contributed equally to this work (co-last authors).

**Table of contents**

[1.	Sample	5](#_Toc222476874)

[2.	Clinical assessment	9](#_Toc222476875)

[3.	Factor analysis of CR	10](#_Toc222476876)

[4.	MRI scanners	11](#_Toc222476877)

[5.	Clinical information of the sample	13](#_Toc222476878)

[6. Demographic and clinical characteristics of the sample subdivided by offspring subgroups.	15](#_Toc222476879)

[7.	Differences in CR across groups and relationship with psychosocial functioning	18](#_Toc222476880)

[8.	Associations between CR and global brain measures with total intracranial volume as a covariate	19](#_Toc222476881)

[9.	Associations between CR and global brain measures with total intracranial volume as a covariate by offspring subgroups	20](#_Toc222476882)

[10.	Sensitivity analyses for the entire sample	26](#_Toc222476883)

[11.	Analyses considering sex	28](#_Toc222476884)

[12.	References	37](#_Toc222476885)

**List of tables**

[**Table S1.** Demographic and clinical comparisons between participants with a single assessment and those with multiple assessments.	7](#_Toc222477590)

[**Table S2.** Relatedness between participants.	8](#_Toc222477591)

[**Table S3.** Factor loading of CR variables on CR model.	10](#_Toc222477592)

[**Table S4.** Distribution of scans performed according to groups at each time point.	11](#_Toc222477593)

[**Table S5.** Intraclass correlation coefficient estimates for lobar cortical surface area inter-site reliability based on absolute agreement and consistency.	12](#_Toc222477594)

[**Table S6.** Clinical information of the sample across all timepoints.	13](#_Toc222477595)

[**Table S7.** Lifetime History of Axis I Disorders across all timepoints.	14](#_Toc222477596)

[**Table S8.** Demographic and clinical characteristics of the sample subdivided by schizophrenia offspring individuals with and without psychotic spectrum symptoms.	15](#_Toc222477597)

[**Table S9.** Demographic and clinical characteristics of the sample subdivided by bipolar disorder offspring individuals with and without psychotic spectrum symptoms.	16](#_Toc222477598)

[**Table S10.** Associations between cognitive reserve and global neuroimaging measures with total intracranial volume as a covariate.	19](#_Toc222477599)

[**Table S11.** Associations between cognitive reserve and global neuroimaging measures with total intracranial volume as a covariate in the schizophrenia offspring subgroup.	20](#_Toc222477600)

[**Table S12.** Associations between cognitive reserve and global neuroimaging measures in the bipolar disorder offspring subgroup.	23](#_Toc222477601)

[**Table S13.** Associations between intelligence quotient and global neuroimaging measures.	27](#_Toc222477602)

**List of figures**

[**Figure S1.** Participants flowchart	5](#_Toc222477603)

[**Figure S2.** Distribution of CR across groups.	18](#_Toc222477604)

[**Figure S3.** Interaction CR-by-group on intracranial volume in the schizophrenia offspring subgroup.	21](#_Toc222477605)

[**Figure S4.** Interaction CR-by-group on intracranial volume in the bipolar disorder offspring subgroup.	24](#_Toc222477606)

[**Figure S5.** CR distribution across groups stratified by sex in the overall sample.	28](#_Toc222477607)

[**Figure S6.** Total intracranial volume distribution across groups stratified by sex in the overall sample.	29](#_Toc222477608)

[**Figure S7.** White matter volume distribution across groups stratified by sex in the overall sample.	30](#_Toc222477609)

[**Figure S8.** CR distribution across schizophrenia offspring subgroups stratified by sex.	31](#_Toc222477610)

[**Figure S9.** Total intracranial volume distribution across schizophrenia offspring subgroups stratified by sex.	32](#_Toc222477611)

[**Figure S10.** White matter volume distribution across schizophrenia offspring subgroups stratified by sex.	33](#_Toc222477612)

[**Figure S11.** CR distribution across bipolar disorder offspring subgroups stratified by sex.	34](#_Toc222477613)

[**Figure S12.** Total intracranial volume distribution across bipolar disorder offspring subgroups stratified by sex.	35](#_Toc222477614)

[**Figure S13.** White matter volume distribution across bipolar disorder offspring subgroups stratified by sex.	36](#_Toc222477615)

# 1. Sample

The study involved a total of 404 data acquisitions from 43 bipolar disorder offspring and schizophrenia offspring who developed psychotic spectrum symptoms over time (SzBpO-P), 94 bipolar disorder offspring and schizophrenia offspring who did not develop psychotic spectrum symptoms over time (SzBpO-NP), and 80 community control offspring (CcO) with multiple assessments across different time points (181 SzBpO-NP, 101 SzBpO-P, 122 CcO).

The analyses within the offspring subgroups included 27 SzO who developed psychotic spectrum symptoms over time (SzO-P) and 24 SzO who did not develop psychotic spectrum symptoms over time (SzO-NP), which yielded 62 datapoints in SzO-P and 39 in SzO-NP. BpO were also divided into 16 who developed psychotic spectrum symptoms over time (BpO-P) and 70 who did not (BpO-NP), yielding 39 datapoints in BpO-P and 142 in BpO-NP.

Figure S1 displays the distribution of participants by group at each time point, as well as those with pending assessments.

**Figure S1.** Participants flowchart

CcO = Offspring of community controls; SzBpO-P = Schizophrenia and bipolar disorder offspring with psychotic spectrum symptoms; SzBpO-NP = Schizophrenia and bipolar disorder offspring without psychotic spectrum symptoms.

a. Single and multiple assessments

To compare timepoint 1 characteristics between participants who completed only one assessment and those who attended multiple follow-ups, mixed models were utilized to examine differences in sociodemographic and clinical variables between these groups. Multilevel mixed-effects logistic regression models were applied for categorical variables, while multilevel mixed-effects linear regression models were used for continuous variables. Group (single assessment or multiple assessments) was included as a fixed factor, and having a sibling in the same study (identified by family number) was included as a random effect. The results of these analyses are presented in Table S1.

No significant differences were found between participants who attended only once and those who attended multiple times in the SzBpO-NP, SzBpO-P, and CcO groups, except for psychosocial functioning in the SzBpO-P group, where participants who attended multiple times demonstrated significantly better psychosocial functioning compared to those who attended only once. In the CcO group, the only exception was the Scale of Prodromal Symptoms (SOPS) general subscale, where participants who attended multiple assessments had significantly lower scores compared to those who attended only once.

No significant differences were found between the groups in the number of families contributing more than one biological offspring to the study. Detailed results of this analysis are presented in Table S2.

**Table S1.** Demographic and clinical comparisons between participants with a single assessment and those with multiple assessments.

|  | **SzBpO-NP** | | | | **SzBpO-P** | | | | **CcO** | | | | |
| --- | --- | --- | --- | --- | --- | --- | --- | --- | --- | --- | --- | --- | --- |
|  | **Single assessment**  **(n = 32)** | **Multiple assessments**  **(n = 62)** | **F** | **P** | **Single assessment**  **(n = 9)** | **Multiple assessments**  **(n = 34)** | **F** | **p** | **Single assessment**  **(n = 32)** | **Multiple assessments**  **(n = 48)** | **F** | **p** |  |
|  | N (%) or Adjusted Mean (SE) | |  |  | N (%) or Adjusted Mean (SE) | |  |  | N (%) or Adjusted Mean (SE) | |  |  |  |
| **Age** | 11.94 (0.58) | 12.46 (0.41) | 0.520 | 0.473 | 11.94 (1.11) | 11.84 (0.57) | 0.006 | 0.937 | 13.27 (1.46) | 13.81 (1.03) | 0.344 | 0.559 |  |
| **Sex:** female | 18 (56.25) | 30 (48.38) | 0.257 | 0.613 | 4 (21.05) | 19 (55.88) | 0.033 | 0.857 | 20 (62.50) | 24 (50.00) | 1.611 | 0.208 |  |
| **CR** | -2.63 (0.55) | -2.15 (0.40) | 0.481 | 0.490 | -3.90 (1.35) | -3.18 (0.68) | 0.387 | 0.547 | 1.54 (0.79) | 1.09 (0.56) | 0.837 | 0.363 |  |
| **Lifetime psychopathology** | 20 (62.50) | 36 (58.06) | 0.172 | 0.679 | 7 (77.77) | 23 (67.64) | 0.341 | 0.562 | 13 (40.62) | 18 (37.50) | 0.630 | 0.430 |  |
| **Psychotropic medication** | 2 (6.25) | 0 (0) | 0.046 | 0.830 | 2 (22.22) | 2 (5.88) | 1.953 | 0.170 | 0 (0) | 0 (0) | NA | NA |  |
| **Substance use** | 3 (9.37) | 1 (1.61) | 0.003 | 0.953 | 1 (11.11) | 1 (2.94) | 0.855 | 0.360 | 2 (6.25) | 3 (6.25) | 2.149 | 0.147 |  |
| **SOPS positive** | 0.79 (0.24) | 0.80 (0.17) | 0.001 | 0.972 | 4.11 (1.03) | 2.16 (0.56) | 2.712 | 0.108 | 0.12 (0.41) | 0.27 (0.27) | 0.232 | 0.632 |  |
| **SOPS negative** | 1.48 (0.45) | 1.11 (0.28) | 1.004 | 0.320 | 3.44 (1.35) | 2.40 (0.74) | 0.459 | 0.503 | 0.40 (0.12) | 0.32 (0.10) | 0.231 | 0.632 |  |
| **SOPS disorganized** | 1.78 (0.40) | 1.41 (0.25) | 1.308 | 0.257 | 1.77 (0.81) | 1.93 (0.44) | 0.028 | 0.869 | 0.57 (0.20) | 0.52 (0.15) | 0.064 | 0.803 |  |
| **SOPS general** | 0.86 (0.29) | 1.03 (0.21) | 0.233 | 0.631 | 3.10 (1.24) | 1.64 (0.65) | 1.714 | 0.233 | 0.71 (0.16) | 0.23 (0.13) | 5.088 | **0.027** |  |
| **SOPS total** | 5.21 (1.15) | 4.51 (0.71) | 0.538 | 0.466 | 11.88 (3.53) | 7.86 (1.93) | 0.999 | 0.324 | 2.34 (0.42) | 1.67 (0.35) | 1.492 | 0.226 |  |
| **GAF** | 77.80 (2.03) | 80.03 (1.43) | 0.804 | 0.372 | 65.55 (4.56) | 76.69 (2.38) | 4.687 | **0.036** | 89.20 (1.38) | 87.04 (1.08) | 1.507 | 0.223 |  |

CcO = Offspring of community controls; CR = Cognitive Reserve; GAF = Global Assessment of Functioning; NA = Not applicable; SE = standard error; SOPS = Scale of Prodromal Symptoms; SzBpO-P = Schizophrenia and bipolar disorder offspring with psychotic spectrum symptoms; SzBpO-NP = Schizophrenia and bipolar disorder offspring without psychotic spectrum symptoms.

b. Relatedness between participants

**Table S2.** Relatedness between participants.

|  | SzBpO-NP | SzBpO-P | CcO | F | p |
| --- | --- | --- | --- | --- | --- |
| **Families contributing more than one biological offspring to the study** | 21 (30.43) | 11 (42.30) | 16 (30.18) | 0.590 | 0.555 |

CcO = Offspring of community controls; SzBpO-P = Schizophrenia and bipolar disorder offspring with psychotic spectrum symptoms; SzBpO-NP = Schizophrenia and bipolar disorder offspring without psychotic spectrum symptoms.

# 3. Clinical assessment

All timepoints included the same clinical, cognitive reserve (CR), and neuroimaging assessments. Children's and adolescents’ lifetime psychopathology was assessed using the Kiddie Schedule for Affective Disorders and Schizophrenia, Present and Lifetime version (K-SADS-PL)[1]. This comprehensive interview was conducted individually to both parents and children during separate interviews. At baseline team members were blinded to each other’s assessment. For participants aged 18 years and older, the Structured Interview for DSM-IV disorders (SCID-I)[2] was employed.

Prodromal psychotic symptoms were evaluated using the Semi-structured Interview for Prodromal Symptoms (SIPS) along with the Scale of Prodromal Symptoms (SOPS).[3] The SOPS consists of 19 items grouped into four subscales: positive, negative, disorganized, and general symptoms. Items are rated on a scale ranging from zero (absent) to 6 (extreme or psychotic intensity). Individuals scoring more than 3 in any item of the positive and negative subscale, were classified as presenting psychotic spectrum symptoms.[4] This classification included both individuals with attenuated psychotic symptoms (scores between 3 and 5), and with psychotic symptoms (score of 6).

In addition, psychosocial functioning was assessed using the Global Assessment of Functioning Scale (GAF),[5] which provides a numeric rating (ranging from 1 to 100) of an individual's social and occupational functioning as well as the severity of psychological symptoms. Higher GAF scores are indicative of better overall functioning.

# 4. Factor analysis of CR

CR was assessed at all the timepoints and was calculated using the same approach used in previous studies conducted by our group with children and adolescents offspring of patients with schizophrenia (SzO) or bipolar disorder (BpO)[6] or diagnosed with first-episode psychosis.[7, 8] Further details about the selected variables included in the CR proxy can be found elsewhere.[6]

The factor analysis was performed exclusively on the CcO group at baseline to ensure a homogeneous estimation of CR. As in our previous research, the factor analysis grouped the seven variables into three factors, accounting for 73.2% of the variance. The Kaiser-Meyer Olkin value was 0.664, exceeding the recommended value of 0.6 (Kaiser, 1970) and Barlett’s Test of Sphericity reached statistical significance (p < 0.001), thus supporting the factorability of the correlation matrix. The analysis revealed the presence of three components explaining 39.34%, 19.46%, and 14.40% of the variance respectively. In the light of previous literature,[6–10] the three factorial scores were added together to obtain a single CR score for each subject. Table S3 shows the results of the CR factor analysis.

**Table S3.** Factor loading of CR variables on CR model.

| **Variable** | **Factor 1** | **Factor 2** | **Factor 3** |
| --- | --- | --- | --- |
| Sociability | 0.969 |  |  |
| Peer relationships | 0.958 |  |  |
| School performance | 0.902 |  |  |
| Level of studies |  | 0.770 |  |
| Questions about the development of language, motor skills and writing and reading |  | 0.817 |  |
| Vocabulary    (WISC-IV or WAIS-IV) |  |  | 0.788 |
| Parents’ socioeconomic status |  |  | 0.677 |

WISC: Wechsler Intelligence Scale for Children; WAIS: Wechsler Adult Intelligence Scale.

The CR score in the SzO and BpO groups at baseline, and at the three groups in other timepoints was calculated by multiplying the z-score of the variables of CR by the coefficients of the component score coefficient matrix of the F factor analysis conducted with CcO at baseline. This approach ensured consistent weighting of the variables across all assessments.

# 4. MRI scanners

A high-resolution T1-weighted 3-dimensional magnetization-prepared rapid sequence was obtained on a 3 Tesla Siemens Magnetom Trio Tim or an upgraded Prisma Fit scanner (Siemens Medical Systems, Germany) at the Centre for Image Diagnosis of the Hospital Clínic, Barcelona, using the following parameters: 240 sagittal slices; TR=2300ms; TE=3.01 ms; slice thickness=1 mm; inversion time(TI)=900; FOV=394x240; matrix size=256×256; flip angle=9°. An inter-scanner reliability study was conducted to assess the compatibility of MRI scans after an in-house scanner upgrade, and these analyses are described also in supplementary material (Table S4). In order to rule out possible underlying pathology, an axial T2 structural image was also acquired and subsequently reviewed by a neuroradiologist blind to group classification. The distribution of cases by MRI scanner is presented in Table 1, with additional details provided in Table S5.

Table S4 shows the distribution of scans performed according to groups at each time point. The analysis for timepoint 5 could not be conducted, as there was only one participant. No differences were observed between groups regarding distribution of scans performed.

**Table S4.** Distribution of scans performed according to groups at each time point.

|  | SzBpO-NP | SzBpO-P | CcO | F | p |
| --- | --- | --- | --- | --- | --- |
|  | N (%) | | |  |  |
| Timepoint 1 |  |  |  | 2.772 | 0.065 |
| Trio | 63 (73.26) | 36 (87.8) | 42 (55.27) |  |  |
| Prisma | 23 (26.74) | 5 (12.20) | 34 (44.73) |  |  |
| Timepoint 2 |  |  |  | 1.751 | 0.179 |
| Trio | 47 (82.46) | 25 (89.29) | 18 (78.26) |  |  |
| Prisma | 10 (17.54) | 3 (10.71) | 5 (21.74) |  |  |
| Timepoint 3 |  |  |  | 1.590 | 0.213 |
| Trio | 22 (75.86) | 14 (87.50) | 15 (93.75) |  |  |
| Prisma | 7 (24.14) | 2 (12.50) | 1 (6.25) |  |  |
| Timepoint 4 |  |  |  | 0.272 | 0.764 |
| Trio | 3 (33.33) | 5 (33.33) | 0 (0) |  |  |
| Prisma | 6 (66.67) | 10 (66.67) | 7 (100.00) |  |  |

CcO = Offspring of community controls; SzBpO-P = Schizophrenia and bipolar disorder offspring with psychotic spectrum symptoms; SzBpO-NP = Schizophrenia and bipolar disorder offspring without psychotic spectrum symptoms.

a. **Inter-scanner reliability study**

A cross-scanner reliability analysis was conducted to evaluate the compatibility between MRI scans acquired from different machines (Trio and Prisma) for lobar cortical measures, including gray matter volume, surface area, and cortical thickness. Reliability refers to the ability to replicate measurements consistently, and it can be assessed by the intra‐class correlation coefficient (ICC).[11] The ICC both quantifies the correlation between measurements and the level of agreement between measurements.[12, 13]

Absolute agreement examines whether different raters assign the same value to the same subjects, whereas consistency assesses whether raters’ values for the same group of subjects are correlated in an additive manner.[12] The ICC ranges from 0 to 1, in which values closer to 1 represent stronger reliability.[12]

Measures of gray matter volume, surface area and cortical thickness measures from all lobes exhibited excellent reliability (ICC > 0.96) for both absolute agreement and consistency, except for insula and cingulate cortex. All the measures of the cingulate lobe showed excellent reliability except for left surface area agreement, that it showed good reliability (ICC > 0.85). For the insula lobe some measures showed moderate reliability (ICC > 0.713). No lobe showed poor reliability (ICC < 0.50). Table S5 summarizes the ICC estimates for surface area, gray matter volume and cortical thickness in each lobe.

**Table S5.** Intraclass correlation coefficient estimates for lobar cortical surface area inter-site reliability based on absolute agreement and consistency.

| Lobe | LH Agreement | LH Consistency | RH Agreement | RH Consistency |
| --- | --- | --- | --- | --- |
| Frontal |  |  |  |  |
| Surface Area | 0.991 | 0.999 | 0.998 | 1.000 |
| Gray matter volume | 0.992 | 0.997 | 0.996 | 0.997 |
| Cortical thickness | 0.982 | 0.977 | 0.953 | 0.940 |
| Cingulate |  |  |  |  |
| Surface Area | 0.850 | 0.924 | 0.980 | 0.974 |
| Gray matter volume | 0.982 | 0.995 | 0.988 | 0.991 |
| Cortical thickness | 0.968 | 0.958 | 0.959 | 0.976 |
| Temporal |  |  |  |  |
| Surface Area | 0.997 | 0.998 | 0.998 | 0.999 |
| Gray matter volume | 0.999 | 0.999 | 0.995 | 0.995 |
| Cortical thickness | 0.979 | 0.973 | 0.883 | 0.902 |
| Parietal |  |  |  |  |
| Surface Area | 0.996 | 0.999 | 0.989 | 0.998 |
| Gray matter volume | 0.996 | 0.995 | 0.989 | 0.997 |
| Cortical thickness | 0.965 | 0.965 | 0.971 | 0.963 |
| Occipital |  |  |  |  |
| Surface Area | 0.989 | 0.985 | 0.983 | 0.978 |
| Gray matter volume | 0.996 | 0.997 | 0.978 | 0.974 |
| Cortical thickness | 0.989 | 0.985 | 0.993 | 0.995 |
| Insula |  |  |  |  |
| Surface Area | 0.737 | 0.713 | 0.732 | 0.843 |
| Gray matter volume | 0.810 | 0.798 | 0.918 | 0.939 |
| Cortical thickness | 0.978 | 0.971 | < 0.70 | < 0.70 |

LH = left hemisphere; RH = right hemisphere.

# 5. Clinical information of the sample

Table S6 presents the results of group comparisons on attenuated psychotic symptoms. The SzBpO-P group exhibited greater clinical severity in all the SOPS subscales, as well in SOPS total, compared to both the SzBpO-NP and the CcO groups.

**Table S6.** Clinical information of the sample across all timepoints.

|  | SzBpO-NP | SzBpO-P | CcO | F | p | SzBpO-P vs. CcO | SzBpO-NP vs. CcO | SzBpO-P vs. SzBpO-NP |
| --- | --- | --- | --- | --- | --- | --- | --- | --- |
|  | N (%) or Adjusted Mean (SE) | | |  |  | Post-hoc^a^ | | |
| **SOPS positive** | 0.60 (0.20) | 2.47 (0.23) | 0.56 (0.23) | 33.803 | **<0.001** | **<0.001** | 1.000 | **<0.001** |
| **SOPS negative** | 0.77 (0.16) | 3.26 (0.22) | 0.51 (0.20) | 51.912 | **<0.001** | **<0.001** | 0.930 | **<0.001** |
| **SOPS disorganized** | 1.11 (0.14) | 2.04 (0.16) | 0.75 (0.16) | 22.103 | **<0.001** | **<0.001** | 0.111 | **<0.001** |
| **SOPS general** | 0.97 (0.14) | 2.13 (0.18) | 0.56 (0.17) | 20.271 | **<0.001** | **<0.001** | 0.202 | **<0.001** |
| **SOPS total** | 3.53 (0.43) | 9.97 (0.57) | 2.47 (0.52) | 54.213 | **<0.001** | **<0.001** | 0.352 | **<0.001** |

Note: All the analyses include subject ID and family membership as random variables. CcO = Offspring of community controls; SE = standard error; SzBpO-P = Schizophrenia and bipolar disorder offspring with psychotic spectrum symptoms; SzBpO-NP = Schizophrenia and bipolar disorder offspring without psychotic spectrum symptoms; SOPS = Scale of Prodromal Symptoms. ^a^Bonferroni multiple comparisons post-hoc.

Table S7 displays group comparisons across different psychiatric diagnoses. No significant differences were observed in the types of psychiatric diagnoses between groups.

**Table S7.** Lifetime History of Axis I Disorders across all timepoints.

|  | SzBpO-NP | SzBpO-P | CcO | F | p | SzBpO-P vs. CcO | | SzBpO-NP vs. CcO | SzBpO-P vs. SzBpO-NP |
| --- | --- | --- | --- | --- | --- | --- | --- | --- | --- |
|  | N (%) | | |  |  | Post-hoc^c^ | | | |
| **Attention-deficit/hyperactivity disorder** | 34 (18.78) | 27 (26.73) | 5 (4.09) | 8.513 | **<0.001** | Ns after Bonferroni correction | | | |
| **Mood disorders ^a^** | 15 (8.28) | 17 (16.83) | 5 (4.09) | 4.088 | **0.018** | Ns after Bonferroni correction | | | |
| **Anxiety disorders ^b^** | 18 (9.94) | 37 (36.63) | 9 (7.37) | 15.889 | **<0.001** | Ns after Bonferroni correction | | | |
| **Oppositional defiant disorder** | 15 (8.28) | 7 (6.93) | 0 (0) | 0.114 | 0.893 |  | | | |
| **Eating disorders** | 4 (2.20) | 3 (2.97) | 0 (0) | 0.094 | 0.910 |  |  | |  |
| **Substance use disorders** | 5 (2.76) | 2 (1.98) | 0 (0) | 0.159 | 0.853 |  |  | |  |

Note: Diagnoses were elicited using DSM-IV criteria (Kiddie-Schedule for Affective Disorders and Schizophrenia for School-Age Children or Structured Interview for DSM-IV disorders (SCID-I). All the analyses include subject ID and family membership as random variables. CcO = Offspring of community controls; SzBpO-P = Schizophrenia and bipolar disorder offspring with psychotic spectrum symptoms; SzBpO-NP = Schizophrenia and bipolar disorder offspring without psychotic spectrum symptoms. ^a^Included major depressive disorder, adjustment disorders, dysthymia, and bipolar disorder. ^b^Included generalised anxiety disorder, simple phobia, panic disorders, separation anxiety disorder, social anxiety, posttraumatic stress disorder, and obsessive-compulsive disorder. ^c^Bonferroni multiple comparisons post-hoc.

# 6. Demographic and clinical characteristics of the sample subdivided by offspring subgroups.

a. **Schizophrenia offspring**

We conducted subanalyses on the CR-by-group interaction on global MRI variables categorizing the sample by offspring subgroups. Regarding schizophrenia offspring (SzO) we divided them into those who developed psychotic spectrum symptoms over time (SzO-P) and those who did not (SzO-NP). This included 27 SzO-P and 24 SzO-NP assessed at multiple time points (62 SzO-P and 39 SzO-NP). Table S11 provides a detailed description of the demographic and clinical characteristics of this subsample.

Significant differences between groups were observed in age, with SzO-NP being significantly younger than controls. No age differences were found between the SzO and CcO groups, nor between the SzO-P and SzO-NP groups. No significant differences were observed between groups in sex, lifetime Axis I diagnosis, psychotropic medication use, substance use, or MRI machine type, after applying Bonferroni correction.

As expected, the SzO-P group exhibited greater severity in attenuated psychotic symptoms, as well as a poorer psychosocial functioning compared to the CcO group. Additionally, the SzO-P group showed significantly greater severity of attenuated psychotic symptoms (positive, negative, general subscales and total score) than the SzO-NP group. The SzO-NP group showed significantly greater severity in attenuated psychotic symptoms on the disorganized subscale, as well as a poorer psychosocial functioning, compared to the CcO group.

The CcO group showed significantly higher levels of CR compared to the SzO-P and SzO-NP groups.

**Table S8.** Demographic and clinical characteristics of the sample subdivided by schizophrenia offspring individuals with and without psychotic spectrum symptoms.

|  | SzO-NP | SzO-P | | CcO | | F | p | SzO-P vs. CcO | SzO-NP vs. CcO | SzO-P vs. SzO-NP |
| --- | --- | --- | --- | --- | --- | --- | --- | --- | --- | --- |
|  | N (%) or Adjusted Mean (SE) | | | | |  |  | Post-hoc^b^ | | |
| **Sociodemographics** |  | |  | |  |  |  |  | | |
| Age | 12.34 (0.59) | | 13.66 (0.46) | | 14.28 (0.33) | 4.160 | **0.017** | 0.840 | **0.013** | 0.240 |
| Sex: female^a^ | 14 (35.89) | | 25 (40.32) | | 99 (42.48) | 1.329 | 0.267 |  |  |  |
| **Clinical** |  | |  | |  |  |  |  | | |
| Psychotropic medication^a^ | 4 (10.25) | | 9 (14.51) | | 1 (0.81) | 4.027 | **0.019** | Ns after Bonferroni correction | | |
| Lifetime axis I diagnosis^a^ | 24 (61.53) | | 43 (69.35) | | 52 (42.62) | 5.986 | **0.003** | Ns after Bonferroni correction | | |
| Substance use^a^ | 2 (5.12) | | 9 (14.51) | | 11 (9.01) | 1.341 | 0.264 |  |  |  |
| SOPS positive | 0.92 (0.35) | | 2.94 (0.28) | | 0.74 (0.21) | 23.453 | **<0.001** | **<0.001** | 1.000 | **<0.001** |
| SOPS negative | 0.75 (0.34) | | 3.39 (0.26) | | 0.51 (0.19) | 39.966 | **<0.001** | **<0.001** | 1.000 | **<0.001** |
| SOPS disorganized | 1.82 (0.28) | | 2.34 (0.22) | | 0.86 (0.19) | 21.800 | **<0.001** | **<0.001** | **0.002** | 0.272 |
| SOPS general | 1.55 (0.35) | | 2.52 (0.29) | | 0.95 (0.26) | 14.128 | **<0.001** | **<0.001** | 0.272 | **0.042** |
| SOPS total | 4.47 (0.94) | | 10.67 (0.73) | | 2.47 (0.51) | 41.772 | **<0.001** | **<0.001** | 0.193 | **<0.001** |
| GAF | 72.47 (2.03) | | 70.99 (1.64) | | 85.55 (1.37) | 43.870 | **<0.001** | **<0.001** | **<0.001** | 1.000 |
| MRI machine model: Trio^a^ | 31 (79.48) | | 48 (77.41) | | 75 (61.47) | 4.301 | **0.015** | Ns after Bonferroni correction | | |
| **CR** | -4.06 (0.54) | | -2.69 (0.43) | | 0.37 (0.34) | 36.767 | **<0.001** | **<0.001** | **<0.001** | 0.116 |

CcO = Offspring of community controls; CR = cognitive reserve; GAF = Global Assessment of Functioning; SE = standard error; SOPS = Scale of Prodromal Symptoms. SzO-P = SzO individuals with psychotic spectrum symptoms; SzO-NP = SzO individuals without psychotic spectrum symptoms. ^a^Multilevel mixed-effect logistic regression models. ^b^Bonferroni multiple comparisons post-hoc.

b. **Bipolar disorder offspring**

Regarding bipolar disorder offspring (BpO) we divided them into those who developed psychotic spectrum symptoms over time (BpO-P) and those who did not (BpO-NP). This included 16 BpO-P and 70 BpO-NP assessed at multiple time points (39 BpO-P and 142 BpO-NP). Table S9 provides a comprehensive overview of the demographic and clinical characteristics of this subsample.

No significant differences were found between groups in terms of age, sex, substance use, MRI machine type, or CR. The BpO-P group exhibited significantly greater severity in attenuated psychotic symptoms, and poorer psychosocial functioning compared to both the CcO and BpO-NP groups. Moreover, no significant differences emerged between the BpO-NP and CcO groups across clinical measures, except for psychosocial functioning, where the BpO-NP group scored significantly lower than the CcO group.

**Table S9.** Demographic and clinical characteristics of the sample subdivided by bipolar disorder offspring individuals with and without psychotic spectrum symptoms.

|  | BpO-NP | BpO-P | | CcO | | F | p | BpO-P vs. CcO | BpO-NP vs. CcO | BpO-P vs. BpO-NP |
| --- | --- | --- | --- | --- | --- | --- | --- | --- | --- | --- |
|  | N (%) or Adjusted Mean (SE) | | | | |  |  | Post-hoc^b^ | | |
| **Sociodemographics** |  | |  | |  |  |  |  | | |
| Age | 16.36 (0.45) | | 16.14 (0.66) | | 15.60 (0.53) | 1.216 | 0.298 |  |  |  |
| Sex: female^a^ | 94 (56.62) | | 23 (50.00) | | 60 (42.48) | 1.089 | 0.338 |  |  |  |
| **Clinical** |  | |  | |  |  |  |  | | |
| Psychotropic medication^a^ | 3 (1.80) | | 8 (17.39) | | 1 (0.81) | 8.828 | **<0.001** | Ns after Bonferroni correction | | |
| Lifetime axis I diagnosis^a^ | 83 (50.00) | | 38 (82.60) | | 52 (42.62) | 9.736 | **<0.001** | **0.013** | 0.198 | 0.078 |
| Substance use^a^ | 17 (10.24) | | 4 (8.69) | | 11 (9.01) | 0.114 | 0.892 |  |  |  |
| SOPS positive | 0.37 (0.18) | | 1.95 (0.27) | | 0.41 (0.21) | 17.855 | **<0.001** | **<0.001** | 1.000 | **<0.001** |
| SOPS negative | 0.70 (0.30) | | 3.51 (0.30) | | 0.51 (0.18) | 38.374 | **<0.001** | **<0.001** | 1.000 | **<0.001** |
| SOPS disorganized | 0.74 (0.09) | | 1.75 (0.18) | | 0.60 (0.11) | 15.507 | **<0.001** | **<0.001** | 0.990 | **<0.001** |
| SOPS general | 0.80 (0.14) | | 2.14 (0.24) | | 0.49 (0.17) | 18.102 | **<0.001** | **<0.001** | 0.329 | **<0.001** |
| SOPS total | 2.83 (0.46) | | 9.55 (0.77) | | 2.26 (0.54) | 38.395 | **<0.001** | **<0.001** | 1.000 | **<0.001** |
| GAF | 84.74 (1.00) | | 73.83 (1.56) | | 87.71 (1.17) | 32.792 | **<0.001** | **<0.001** | **0.042** | **<0.001** |
| MRI machine model: Trio^a^ | 119 (71.68) | | 37 (80.43) | | 75 (61.47) | 1.373 | 0.255 |  | | |
| **CR** | 0.52 (0.34) | | 0.10 (0.53) | | 0.90 (0.41) | 1.050 | 0.351 |  |  |  |

BpO-NP = BpO individuals without psychotic spectrum symptoms; BpO-P = BpO individuals with psychotic spectrum symptoms; CcO = Offspring of community controls; CR = cognitive reserve; GAF = Global Assessment of Functioning; SE = standard error; SOPS = Scale of Prodromal Symptoms. ^a^Multilevel mixed-effect logistic regression models. ^b^Bonferroni multiple comparisons post-hoc.

# 7. Differences in CR across groups and relationship with psychosocial functioning

**Figure S2.** Distribution of CR across groups.

CcO = Offspring of community controls; CR = Cognitive reserve; SzBpO-P = Schizophrenia and bipolar disorder offspring with psychotic spectrum symptoms; SzBpO-NP = Schizophrenia and bipolar disorder offspring without psychotic spectrum symptoms.

In order to assess the role of CR on psychosocial functioning, analysis including a group-by-CR on psychosocial functioning was conducted with age and sex as covariates and the same random variables (family relatedness and subject ID). The analysis revealed no significant CR-by-group interaction for the whole sample (F = 0.400; p = 0.671). However, higher CR was significantly associated with better psychosocial functioning (F = 77.403; p < 0.001). For the sake of completeness, this was tested for each group individually. In the SzBpO-P group, a significant association between CR and GAF was observed (F = 20.320; p < 0.001). In the SzBpO-NP group, a significant association between CR and GAF was observed (F = 43.554; p < 0.001). In the CcO group, a significant association between CR and GAF was observed (F = 20.374; p < 0.001).

# 8. Associations between CR and global brain measures with total intracranial volume as a covariate

a. **The entire sample**

Intracranial volume was included as a covariate in the analyses of global neuroimaging measures, except for cortical thickness. No significant interactions were found between CR and group for any of the global brain measures. The detailed results of these analyses are presented in Table S10.

**Table S10.** Associations between cognitive reserve and global neuroimaging measures with total intracranial volume as a covariate.

|  | SzBpO-NP | SzBpO-P | CcO | CR | | Group | | CR × group | |
| --- | --- | --- | --- | --- | --- | --- | --- | --- | --- |
|  | Adjusted Mean (SE) | | | F | p | F | p | F | p |
| Total GMV (cm^3^) | 735.47 (2.98) | 731.52 (3.54) | 736.66 (3.38) | 0.320 | 0.572 | 0.663 | 0.516 | 1.996 | 0.137 |
| Total WMV (cm^3^) | 433.79 (1.70) | 432.27 (2.37) | 429.51 (2.21) | 1.666 | 0.198 | 1.239 | 0.291 | 1.079 | 0.341 |
| SA L (cm^2^) | 901.40 (2.89) | 894.02 (4.02) | 904.97 (3.72) | 0.613 | 0.434 | 1.147 | 0.319 | 2.980 | 0.052 |
| SA R (cm^2^) | 896.26 (2.83) | 890.82 (3.94) | 899.96 (3.65) | 0.178 | 0.673 | 0.881 | 0.415 | 2.763 | 0.064 |
| CTH L (cm) | 0.269 (0.001) | 0.268 (0.001) | 0.268 (0.001) | 1.347 | 0.246 | 0.840 | 0.432 | 0.529 | 0.590 |
| CTH R (cm) | 0.268 (0.001) | 0.267 (0.001) | 0.267 (0.001) | 0.721 | 0.396 | 0.333 | 0.717 | 1.254 | 0.287 |

CcO = Offspring of community controls; CR = Cognitive reserve; GMV = Gray Matter Volume; SA = Surface Area; SzBpO-P = Schizophrenia and bipolar disorder offspring with psychotic spectrum symptoms; SzBpO-NP = Schizophrenia and bipolar disorder offspring without psychotic spectrum symptoms; SE = standard error; WMV = White Matter Volume.

# 9. Associations between CR and global brain measures with total intracranial volume as a covariate by offspring subgroups

b. **Schizophrenia offspring**

In SzO, there was a significant CR-by-group interaction on total intracranial volume (F = 7.583; p = 0.001). In CcO (β = 17.462; 95% CI = 5.69 to 29.22; p = 0.004) and SzO-NP (β = 23.145; 95% CI = 10.43 to 35.85; p < 0.001) a positive association was observed, whereby higher levels of CR were associated with larger total intracranial volume compared to the SzBpO-P group. No significant differences were found between the SzO-NP and CcO groups. The detailed results of these analyses are presented in Table S11. No other significant CR-by-group interactions were found in global brain structural measures. These results are graphically represented in Figure S3.

**Table S11.** Associations between cognitive reserve and global neuroimaging measures with total intracranial volume as a covariate in the schizophrenia offspring subgroup.

|  | SzO-NP | SzO-P | | CcO | | CR | | Group | | Post-hoc^a^ | | | CR × group | |  | | |
| --- | --- | --- | --- | --- | --- | --- | --- | --- | --- | --- | --- | --- | --- | --- | --- | --- | --- |
|  | Adjusted Mean (SE) | | | | | F | p | F | p | SzO-P vs. CcO | SzO-NP vs. CcO | SzO-P vs. SzO-NP | F | p | SzO-P vs. CcO | SzO-NP vs. CcO | SzO-P vs. SzO-NP |
| TIV (cm^3^) | 1590.60 (34.60) | | 1471.40 (27.13) | | 1562.20 (23.80) | 2.055 | 0.153 | 15.484 | **<0.001** | **<0.001** | 0.903 | **<0.001** | 7.583 | **0.001*** | **0.004** | 0.390 | **<0.001** |
| Total GMV (cm^3^) | 740.03 (7.02) | | 737.53 (5.62) | | 743.10 (4.76) | 0.159 | 0.691 | 0.308 | 0.735 |  |  |  | 1.607 | 0.203 |  |  |  |
| Total WMV (cm^3^) | 446.37 (4.51) | | 431.84 (3.32) | | 430.70 (2.40) | 0.108 | 0.743 | 5.487 | **0.005*** | 1.000 | **0.006** | **0.027** | 3.628 | **0.028** | 0.234 | 0.111 | **0.008** |
| SA L (cm^2^) | 916.40 (9.25) | | 899.37 (7.45) | | 917.28 (6.30) | 0.069 | 0.793 | 2.643 | 0.074 |  |  |  | 1.914 | 0.150 |  |  |  |
| SA R (cm^2^) | 910.59 (8.89) | | 895.14 (7.13) | | 910.22 (5.98) | 0.045 | 0.831 | 1.913 | 0.150 |  |  |  | 1.650 | 0.195 |  |  |  |
| CTH L (cm) | 0.267 (0.001) | | 0.266 (0.001) | | 0.267 (0.001) | 1.371 | 0.243 | 0.2787 | 0.751 |  |  |  | 0.038 | 0.963 |  |  |  |
| CTH R (cm) | 0.266 (0.002) | | 0.266 (0.001) | | 0.266 (0.001) | 0.078 | 0.780 | 0.009 | 0.991 |  |  |  | 0.586 | 0.558 |  |  |  |

CcO = Offspring of community controls; CR = Cognitive reserve; CTH = Cortical Thickness; GMV = Gray Matter Volume; SA = Surface Area; SE = standard error; SzO-NP = SzO individuals without psychotic spectrum symptoms; SzO-P = SzO individuals with psychotic spectrum symptoms; WMV = White Matter Volume. *p-values surviving p<.05 (false discovery rate correction).

**Figure S3.** Interaction CR-by-group on intracranial volume in the schizophrenia offspring subgroup.

CcO = Offspring of community controls; SzO-NP = SzO individuals without psychotic spectrum symptoms; SzO-P = SzO individuals with psychotic spectrum symptoms; TIV = Total Intracranial Volume.

c. **Bipolar disorder offspring**

In BpO, a significant CR-by-group interaction was observed in total intracranial volume (F = 5.142; p = 0.006). In, CcO (β = 23.512; 95% CI = 8.75 to 38.27; p = 0.002) and BpO-NP (β = 19.922; 95% CI = 6.53 to 33.31; p = 0.004) a positive association was observed, whereby higher levels of CR were associated with larger total intracranial volume compared to the SzBpO-P group. No significant differences were found between the BpO-NP and CcO groups. No further significant CR-by-group interactions were found in the other global brain structural measures. These results are presented in Table S12 and graphically represented in Figure S4.

**Table S12.** Associations between cognitive reserve and global neuroimaging measures in the bipolar disorder offspring subgroup.

|  | BpO-NP | BpO-P | CcO | CR | | Group | | Post-hoc^a^ | | | CR × group | |  |  |  |
| --- | --- | --- | --- | --- | --- | --- | --- | --- | --- | --- | --- | --- | --- | --- | --- |
|  | Adjusted Mean (SE) | | | F | p | F | p | BpO-P vs. CcO | BpO-NP vs. CcO | BpO-P vs. BpO-NP | F | p | BpO-P vs. CcO | BpO-NP vs. CcO | BpO-P vs. BpO-NP |
| TIV (cm^3^) | 1510.37 (17.37) | 1482.96 (22.20) | 1527.82 (18.11) | 3.585 | 0.059 | 2.138 | 0.120 |  |  |  | 5.142 | **0.006*** | **0.002** | 0.426 | **0.004** |
| Total GMV (cm^3^) | 730.99 (2.63) | 722.80 (4.40) | 730.73 (2.89) | 0.405 | 0.525 | 1.564 | 0.211 |  |  |  | 0.115 | 0.891 |  |  |  |
| Total WMV (cm^3^) | 435.11 (2.44) | 438.57 (3.84) | 431.68 (2.62) | 0.018 | 0.892 | 1.597 | 0.204 |  |  |  | 0.090 | 0.914 |  |  |  |
| SA L (cm^2^) | 900.47 (3.30) | 898.81 (6.08) | 901.41 (3.70) | 1.167 | 0.281 | 0.165 | 0.848 |  |  |  | 1.930 | 0.147 |  |  |  |
| SA R (cm^2^) | 894.58 (3.21) | 892.84 (5.92) | 896.49 (3.60) | 1.314 | 0.253 | 0.288 | 0.750 |  |  |  | 2.063 | 0.129 |  |  |  |
| CTH L (cm) | 0.269 (0.001) | 0.268 (0.001) | 0.268 (0.001) | 5.054 | **0.025** | 0.728 | 0.484 |  |  |  | 2.565 | 0.079 |  |  |  |
| CTH R (cm) | 0.268 (0.001) | 0.266 (0.002) | 0.267 (0.001) | 3.225 | 0.073 | 0.703 | 0.496 |  |  |  | 2.272 | 0.105 |  |  |  |

CcO = Offspring of community controls; BpO-NP = BpO individuals without psychotic spectrum symptoms; BpO-P = BpO individuals with psychotic spectrum symptoms; CR = Cognitive reserve; CTH = Cortical Thickness; GMV = Gray Matter Volume; TIV = Total Intracranial Volume; SA = Surface Area; SE = standard error; SzO = Offspring of patients with schizophrenia; TIV = Total Intracranial Volume; WMV = White Matter Volume. *p-values surviving p<.05 (false discovery rate correction). ^a^Bonferroni multiple comparisons post-hoc. Note: age, sex and MRI machine included as covariable in all the analyses.

**Figure S4.** Interaction CR-by-group on intracranial volume in the bipolar disorder offspring subgroup.

CcO = Offspring of community controls; BpO-NP = BpO individuals without psychotic spectrum symptoms; BpO-P = BpO individuals with psychotic spectrum symptoms; TIV = Total Intracranial Volume.

# 10. Sensitivity analyses for the entire sample

a. Premorbid intelligence quotient

In order to assess if IQ showed a stronger association with global brain measures compared to CR, we repeated the analysis with IQ instead of CR. These analyses were also conducted because IQ is the only non-modifiable factor of CR. Thus, we aimed to assess the influence of modifiable versus non-modifiable factors in their association with brain measures. For these analyses, the vocabulary score from the WISC-IV or WAIS-IV was used as a measure of premorbid IQ. Results of these analyses are presented in Table S13.

In the SzBpO-P group, a negative association was observed, where a higher IQ was associated with lower total intracranial volume compared to the CcO (β = -6.886; 95% CI = -12.34 to -1.43; p = 0.013) and the SzBpO-NP (β = -12.909; 95% CI = -20.55 to -5.26; p = 0.001) groups. No significant differences were found between the CcO and SzBpO-NP groups.

The same pattern of group differences was observed in the association between IQ and total white matter volume. Again, the SzBpO-P group showed a negative association compared to both the CcO (β = -3.15; 95% CI = -5.12 to -1.18; p = 0.002) and the SzBpO-NP (β = -5.178; 95% CI = -7.92 to -2.43; p < 0.001) groups. No significant differences were found between the SzBpO-NP and CcO groups.

**Table S13.** Associations between intelligence quotient and global neuroimaging measures.

|  | SzBpO-NP | SzBpO-P | CcO | IQ | | Group | | Post-hoc^a^ | | | IQ × group | |  |  |  |
| --- | --- | --- | --- | --- | --- | --- | --- | --- | --- | --- | --- | --- | --- | --- | --- |
|  | Adjusted Mean (SE) | | | F | p | F | p | SzBpO-P vs. CcO | SzBpO-NP vs. CcO | SzBpO-P vs. SzBpO-NP | F | p | SzBpO-P vs. CcO | SzBpO-NP vs. CcO | SzBpO-P vs. SzBpO-NP |
| TIV (cm^3^) | 1531.04 (17.29) | 1481.04 (18.37) | 1549.21 (17.75) | 1.140 | 0.286 | 2.947 | 0.054 |  |  |  | 5.767 | **0.003*** | **0.013** | 0.064 | **0.001** |
| Total GMV (cm^3^) | 731.49 (7.44) | 707.90 (7.92) | 739.74 (7.65) | 1.761 | 0.185 | 2.752 | 0.065 |  |  |  | 2.436 | 0.089 |  |  |  |
| Total WMV (cm^3^) | 428.90 (5.77) | 412.61 (6.24) | 429.66 (5.96) | 0.568 | 0.452 | 3.439 | **0.033** | **0.007** | 1.000 | **0.005** | 7.662 | **0.001*** | **0.002** | 0.083 | **<0.001** |
| SA L (cm^2^) | 898.64 (9.06) | 861.51 (9.99) | 910.35 (9.45) | 2.487 | 0.116 | 2.599 | 0.076 |  |  |  | 2.939 | 0.054 |  |  |  |
| SA R (cm^2^) | 890.56 (9.31) | 856.33 (10.13) | 902.44 (9.65) | 1.740 | 0.188 | 2.495 | 0.084 |  |  |  | 3.185 | **0.042** | 0.141 | 0.080 | **0.012** |
| CTH L (cm) | 0.269 (0.001) | 0.268 (0.001) | 0.268 (0.001) | 0.016 | 0.899 | 0.338 | 0.713 |  |  |  | 0.461 | 0.631 |  |  |  |
| CTH R (cm) | 0.268 (0.001) | 0.268 (0.001) | 0.268 (0.001) | 0.032 | 0.859 | 0.388 | 0.679 |  |  |  | 0.441 | 0.644 |  |  |  |

CcO = Offspring of community controls; CTH = Cortical Thickness; GMV = Gray Matter Volume; SzBpO-P = Schizophrenia and bipolar disorder offspring with psychotic spectrum symptoms; SzBpO-NP = Schizophrenia and bipolar disorder offspring without psychotic spectrum symptoms; TIV = Total Intracranial Volume; IQ = intelligence quotient; SA = Surface Area; SE = standard error; WMV = White Matter Volume. *p-values surviving p<.05 (false discovery rate correction). ^a^Bonferroni multiple comparisons post-hoc. Note: age, sex and MRI machine included as covariable in all the analyses.

# 11. Analyses considering sex

Sex was included as a covariate in all analyses, and groups were matched for both age and sex. To explore whether sex influenced the interaction CR × group on intracranial volume and white matter volume, we conducted analyses that included a three-way interaction (CR × group × sex). This interaction was not significant both for intracranial volume (F = 1.464; p = 0.233) and for white matter volume (F = 1.754; p = 0.174), suggesting that sex may not play a role in modulating the association between CR and intracranial and white matter volumes across groups.

Nevertheless, for informational purposes, we provide figures displaying the distribution of CR, intracranial volume and white matter volume across groups (SzBpO-P, SzBpO-NP, and CcO) and across offspring subgroups (SzO-P, SzO-NP, BpO-P, and BpO-NP) stratified by sex.

a. **Overall sample**

**Figure S5.** CR distribution across groups stratified by sex in the overall sample.

CcO = Offspring of community controls; CR = Cognitive reserve; SzBpO-P = Schizophrenia and bipolar disorder offspring with psychotic spectrum symptoms; SzBpO-NP = Schizophrenia and bipolar disorder offspring without psychotic spectrum symptoms.

**Figure S6.** Total intracranial volume distribution across groups stratified by sex in the overall sample.

CcO = Offspring of community controls; SzBpO-P = Schizophrenia and bipolar disorder offspring with psychotic spectrum symptoms; SzBpO-NP = Schizophrenia and bipolar disorder offspring without psychotic spectrum symptoms; TIV = Total intracranial volume.

**Figure S7.** White matter volume distribution across groups stratified by sex in the overall sample.

CcO = Offspring of community controls; SzBpO-P = Schizophrenia and bipolar disorder offspring with psychotic spectrum symptoms; SzBpO-NP = Schizophrenia and bipolar disorder offspring without psychotic spectrum symptoms; TIV = Total intracranial volume; WMV = White matter volume.

b. **Schizophrenia offspring**

**Figure S8.** CR distribution across schizophrenia offspring subgroups stratified by sex.

CR = Cognitive reserve; SzO-NP = SzO individuals without psychotic spectrum symptoms; SzO-P = SzO individuals with psychotic spectrum symptoms.

**Figure S9.** Total intracranial volume distribution across schizophrenia offspring subgroups stratified by sex.

SzO-NP = SzO individuals without psychotic spectrum symptoms; SzO-P = SzO individuals with psychotic spectrum symptoms; TIV = Total intracranial volume.

**Figure S10.** White matter volume distribution across schizophrenia offspring subgroups stratified by sex.

SzO-NP = SzO individuals without psychotic spectrum symptoms; SzO-P = SzO individuals with psychotic spectrum symptoms; WMV = White matter volume.

c. **Biploar disorder offspring**

**Figure S11.** CR distribution across bipolar disorder offspring subgroups stratified by sex.

BpO-NP = BpO individuals without psychotic spectrum symptoms; BpO-P = BpO individuals with psychotic spectrum symptoms; CR = Cognitive reserve.

**Figure S12.** Total intracranial volume distribution across bipolar disorder offspring subgroups stratified by sex.

BpO-NP = BpO individuals without psychotic spectrum symptoms; BpO-P = BpO individuals with psychotic spectrum symptoms; TIV = Total intracranial volume.

**Figure S13.** White matter volume distribution across bipolar disorder offspring subgroups stratified by sex.

BpO-NP = BpO individuals without psychotic spectrum symptoms; BpO-P = BpO individuals with psychotic spectrum symptoms; WMV = White matter volume.

# 12. References

1.	Kaufman J, Birmaher B, Brent D, et al (1997) Schedule for affective disorders and schizophrenia for school-age children-present and lifetime version (K-SADS-PL): Initial reliability and validity data. J Am Acad Child Adolesc Psychiatry 36:980–988

2.	First M, Spitzer R, Gibbon M, Williams J (1997) User’s guide for the Structured clinical interview for DSM-IV axis I disorders SCID-I: clinician version

3.	Miller TJ, McGlashan TH, Rosen JL, et al (2002) Prospective diagnosis of the initial prodrome for schizophrenia based on the structured interview for prodromal syndromes: Preliminary evidence of interrater reliability and predictive validity. American Journal of Psychiatry 159:863–865. https://doi.org/10.1176/appi.ajp.159.5.863

4.	Lencz T, Smith CW, Auther A, et al (2004) Nonspecific and attenuated negative symptoms in patients at clinical high-risk for schizophrenia. Schizophr Res 68:37–48. https://doi.org/10.1016/S0920-9964(03)00214-7

5.	Endicott J, Spitzer RL, Fleiss JL, Cohen J (1976) The Global Assessment Scale: A Procedure for Measuring Overall Severity of Psychiatric Disturbance. Arch Gen Psychiatry 33:766–771

6.	Camprodon-Boadas P, Rosa-Justicia M, Sugranyes G, et al (2023) Cognitive reserve and its correlates in child and adolescent offspring of patients diagnosed with schizophrenia or bipolar disorder. Eur Child Adolesc Psychiatry 32:1463–1473. https://doi.org/10.1007/S00787-022-01957-0

7.	Camprodon-Boadas P, de la Serna E, Baeza I, et al (2020) Cognitive reserve in patients with first-episode psychosis as outcome predictor at 5-year follow-up. Eur Child Adolesc Psychiatry. https://doi.org/10.1007/s00787-020-01668-4

8.	de la Serna E, Andrés-Perpiñá S, Puig O, et al (2013) Cognitive reserve as a predictor of two year neuropsychological performance in early onset first-episode schizophrenia. Schizophr Res 143:125–31

9.	Amoretti S, Bernardo M, Bonnin CM, et al (2016) The impact of cognitive reserve in the outcome of first-episode psychoses: 2-year follow-up study. Eur Neuropsychopharmacol 26:1638–48

10.	Amoretti S, Cabrera B, Torrent C, et al (2018) Cognitive reserve as an outcome predictor: first-episode affective versus non-affective psychosis. Acta Psychiatr Scand 138:441–455

11.	Shrout PE, Fleiss JL (1979) Intraclass correlations: uses in assessing rater reliability. Psychol Bull 86:420–428. https://doi.org/10.1037//0033-2909.86.2.420

12.	Koo TK, Li MY (2016) A Guideline of Selecting and Reporting Intraclass Correlation Coefficients for Reliability Research. J Chiropr Med 15:155. https://doi.org/10.1016/J.JCM.2016.02.012

13.	Bruton A, Conway JH, Holgate ST (2000) Reliability: What is it, and how is it measured? Physiotherapy 86:94–99. https://doi.org/10.1016/S0031-9406(05)61211-4
